# Combined assessment of FLAIR vascular hyperintensities and cortical venous outflow for outcome prediction in acute middle cerebral artery occlusion

**DOI:** 10.3389/fnhum.2026.1953695

**Published:** 2026-09-29

**Authors:** Ping Yang, Lingjia Xu, Feng Zhou

**Affiliations:** 1 Department of Endocrinology, Shaoxing Second Hospital, The Second Affiliated Hospital of Shaoxing University, Shaoxing, Zhejiang, China; 2 Department of Neurology, Shaoxing Second Hospital, The Second Affiliated Hospital of Shaoxing University, Shaoxing, Zhejiang, China

**Keywords:** acute ischemic stroke, fluid-attenuated inversion recovery vascular hyperintensities, magnetic resonance imaging, middle cerebral artery, venous outflow

## Abstract

**Background:**

Fluid-attenuated inversion recovery vascular hyperintensities (FVHs) are an imaging marker of leptomeningeal collateral compensation, but their prognostic value in stroke remains controversial. Venous outflow (VO) dysfunction may offset collateral benefits, suggesting that VO status could modulate the prognostic effect of FVHs. In this study, we combined the assessment of FVHs and VO in patients with acute middle cerebral artery (MCA) occlusion who did not receive reperfusion treatment, to determine whether the arteriovenous coupling state predicts 90-day good outcome.

**Methods:**

A total of 152 patients were enrolled and divided into four groups based on FVHs and VO status: Type I (good coupling), Type II (arteriovenous mismatch), Type III (hypoperfusion), and Type IV (arteriovenous failure). G02 was a binary indicator of arteriovenous mismatch (Type II vs. non-Type II), derived from the four-group classification. Multivariable logistic regression was used to analyze the independent association between the four groups and 90-day good outcome. A prediction model was constructed with a baseline model including National Institutes of Health Stroke Scale (NIHSS) score, diffusion-weighted imaging (DWI) volume, and age, and a full model adding G02 to the baseline model. Model performance was evaluated using 5-fold cross-validation with AUC, calibration curves, and decision curves.

**Results:**

Of the 152 patients, 28 (18.4%) were classified as Type II, which was independently associated with reduced odds of good 90-day outcome (*OR* = 0.041, 95%*CI* 0.003 ~ 0.506). Adding G02 to the baseline model (NIHSS + DWI volume + age) improved the AUC from 0.759 to 0.780 (NRI = 0.0305, IDI = 0.0348), and the full model showed greater net benefit on decision curve analysis.

**Conclusion:**

Our findings suggest that impaired VO may offset the favorable effect of collateral compensation reflected by FVHs. In non-reperfused acute MCA occlusion, arteriovenous mismatch independently predicts poor prognosis. Adding this marker to conventional clinical variables modestly improves prognostic performance and provides greater net clinical benefit, supporting combined arterial and venous assessment for stroke risk stratification.

## 1 Introduction

Stroke is a leading cause of death and long-term disability worldwide, with the highest burden observed in low- and middle-income countries (GBD 2019 Stroke Collaborators, 2021). Among ischemic stroke subtypes, large artery occlusion is associated with the poorest outcomes and the highest rates of dependency (Chen et al., 2024). Although revascularization improves outcomes when initiated early, nearly 49% of patients in China present beyond the treatment window and are therefore at increased risk of poor prognosis (Yuan et al., 2023). Early identification of patients who present beyond the time window and are at high risk for poor outcomes is critical for guiding more aggressive medical or elective surgical therapy. Collateral circulation is a key determinant of penumbral viability and clinical outcome (Vagal et al., 2018).

Fluid-attenuated inversion recovery vascular hyperintensities (FVHs) are a commonly used imaging marker of leptomeningeal collateral flow and may reflect slow or stagnant collateral circulation (Lee et al., 2022). Previous studies have confirmed that more abundant FVHs generally indicate better collateral status and are associated with a smaller infarct core and favorable prognosis (Xin et al., 2024; Xiang et al., 2023). However, growing evidence suggests that relying solely on arterial collateral assessment for prognosis prediction has inherent limitations, and that the prognostic value of FVHs may be modulated by other factors (Zhou et al., 2020; Wang et al., 2024).

The role of the venous drainage system in stroke prognosis has gained increasing attention in recent years (van Horn et al., 2022; Li et al., 2023; Favruzzo et al., 2025). The cerebral venous system not only clears metabolic waste but also actively contributes to hemodynamic regulation (Barghash et al., 2025). Arterial collaterals deliver forward flow, while venous outflow (VO) determines drainage patency; together, they shape the recirculation pattern in the ischemic territory (Zhang et al., 2017). Impaired VO can lead to venous hypertension, microcirculatory stasis, and aggravated tissue edema, which may ultimately offset the benefits of arterial collateral flow (Kelani et al., 2025; Li et al., 2025). We hypothesized that VO status may modulate the prognostic value of FVHs. When VO is preserved, FVHs reflect effective collateral compensation and are associated with favorable outcomes. When VO is impaired, collateral flow cannot drain effectively and may instead exacerbate microcirculatory stasis, leading to poor prognosis.

To test this hypothesis, we evaluated FVHs together with VO status in patients with acute middle cerebral artery (MCA) occlusion who did not receive reperfusion therapy. We constructed a predictive model incorporating conventional clinical variables and an arteriovenous mismatch marker (abundant FVHs with impaired VO) to assess its value for 90-day outcome prediction.

## 2 Materials and methods

### 2.1 Participants

This retrospective study included 152 patients with acute MCA occlusion who were hospitalized in the Department of Neurology at Shaoxing Second Hospital from January 2018 to December 2024. Inclusion criteria were as follows: (1) age 18 years or older; (2) time from admission to baseline imaging within 24 h; (3) computed tomography angiography (CTA) confirmation of unilateral M1 or M2 segment occlusion of MCA; (4) no receipt of any reperfusion therapy, including intravenous thrombolysis or mechanical thrombectomy; and (5) availability of complete imaging data. Exclusion criteria were as follows: (1) tandem occlusion; (2) other clearly identified causes such as arterial dissection or vasculitis; (3) prior stroke with residual disability [mRs (modified Rankin Scale score) ≥ 2]; (4) poor magnetic resonance imaging (MRI) image quality; (5) concurrent intracranial venous sinus thrombosis or cortical vein infarction; and (6) concurrent intracranial tumors or other severe systemic diseases that made patients unsuitable for the study. The research was approved by the hospital’s ethics committee. The flow diagram of enrollment of patients was shown in Figure 1.

**Figure 1:**
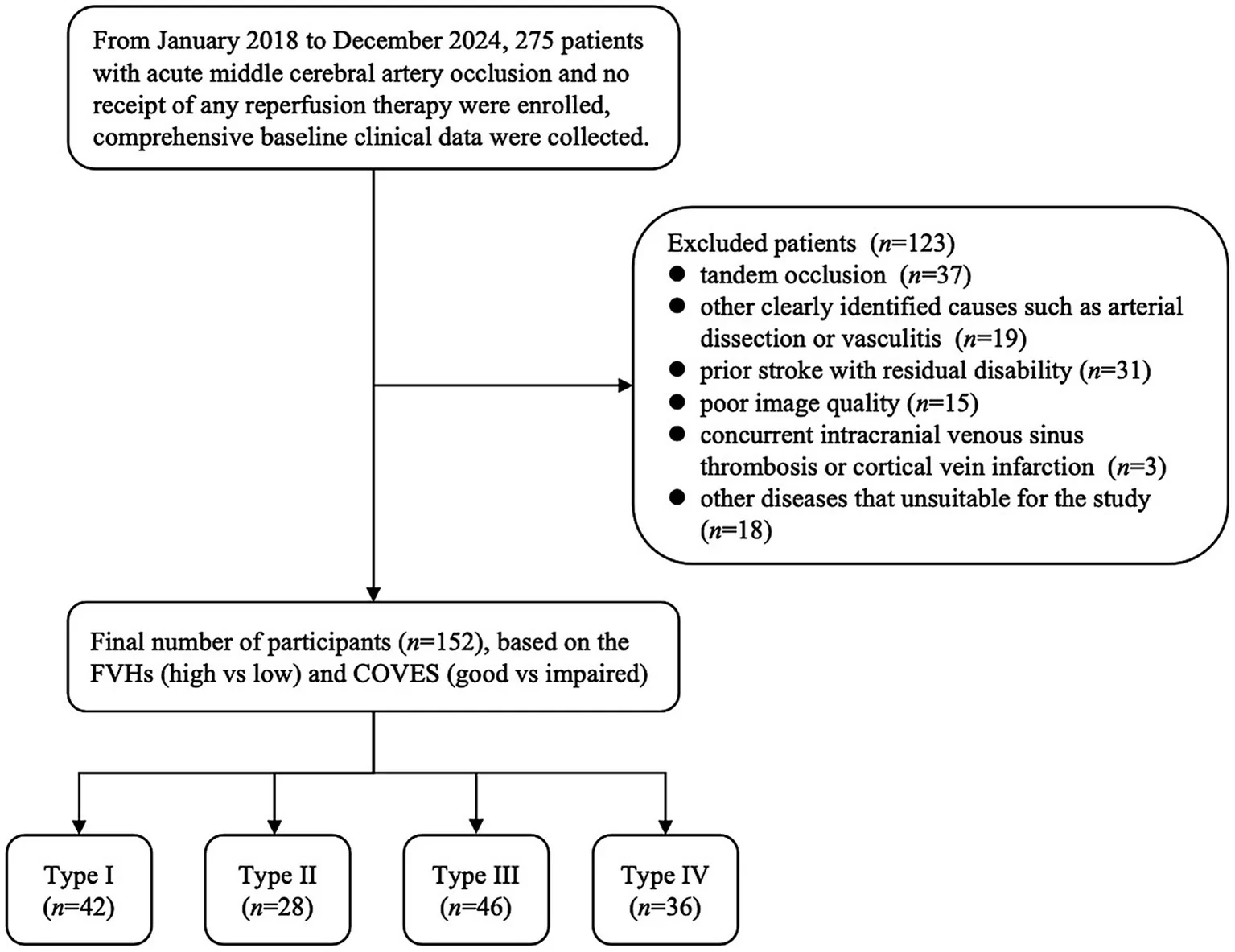
Flow diagram of enrollment of study patients.

### 2.2 Clinical data

Comprehensive baseline clinical data were collected for each patient, including age, sex, vascular risk factors (hypertension, diabetes mellitus, atrial fibrillation, coronary artery disease, prior stroke, and smoking history), admission systolic and diastolic blood pressure, and the time interval from admission to MRI examination. Neurological severity on admission was assessed using the National Institutes of Health Stroke Scale (NIHSS). All imaging evaluations were performed by trained readers who were blinded to clinical outcomes. Laboratory parameters obtained on admission included white blood cell count, neutrophil count, lymphocyte count, neutrophil-to-lymphocyte ratio (NLR), hemoglobin, platelet count, high-sensitivity C-reactive protein (hs-CRP), D-dimer, blood glucose, glycated hemoglobin (HbA1c), total cholesterol (TC), triglycerides (TG), high-density lipoprotein (HDL), low-density lipoprotein (LDL), creatinine (Cr), creatinine clearance rate (Ccr), and homocysteine (Hcy). Basic imaging data were reviewed for the location of arterial stenosis or occlusion and Alberta Stroke Program Early CT Score (ASPECTS). Treatment information was also documented, including use of mannitol, antiplatelet therapy, statins, and decompressive craniectomy. Clinical outcomes at 90 days were assessed using the mRS. A favorable outcome was defined as mRS 0 to 2, and an unfavorable outcome as mRS 3 to 6. The primary endpoint was a good prognosis at 90 days (mRS 0 ~ 2), which was obtained through outpatient clinic visits or standardized telephone interviews.

### 2.3 Imaging protocol and analysis

#### 2.3.1 Imaging protocol

All patients underwent MRI on a 1.5T Siemens MAGNETOM Avanto system (Siemens Healthineers, Erlangen, Germany). The protocol included T1-weighted, T2-weighted, T2-FLAIR, and diffusion-weighted imaging (DWI) sequences. An additional b0 image was obtained without diffusion weighting. Detailed parameters for all sequences are provided in Supplementary Table S1.

Non-contrast head CT and CTA were performed using a 64-slice CT scanner (SOMATOM Definition AS, Siemens Healthineers). Non-contrast CT parameters were set as follows: tube voltage 120 kV, automatic tube current modulation (reference 300 mAs), slice thickness 5 mm, and slice interval 5 mm. For CTA, 50 mL of non-ionic iodinated contrast medium was administered through the right antecubital vein using a power injector at a rate of 5 mL/s, followed by a 30-mL saline flush at the same rate. Scanning was performed with a 5-s delay over a 160-mm range from the skull base to the vertex. CTA images were reconstructed using the integrated CT workstation.

#### 2.3.2 Imaging assessment

All imaging evaluations were performed by two trained researchers who were blinded to clinical outcomes. Inter-observer agreement was assessed using Cohen’s kappa coefficient with 95% confidence intervals (*CI*) for both FVHs grading and COVES scoring, based on a randomly selected subset of 20% of cases (*n* = 30). The kappa values were 0.850 (95% *CI* 0.762 ~ 0.913) for FVHs grading and 0.782 (95% *CI* 0.681 ~ 0.884) for COVES scoring. Disagreements were resolved by consensus.

FVHs were defined as serpentine, tubular, or punctate hyperintensities coursing along the cortical surface on FLAIR images, typically localized distal to the site of arterial occlusion or stenosis. The Lee score was used to grade FVHs as follows: 0 = no vascular hyperintensity; 1 = hyperintensities confined to the distal MCA branches (M4 segment) involving 1 ~ 2 slices; 2 = hyperintensities extending to the proximal-middle MCA segments (M2 ~ M3) without reaching the insular cortex; 3 = hyperintensities widely distributed, including the insular cortex and more proximal regions (Lee et al., 2009). A score of 2 or higher was defined as “high” FVHs.

VO was evaluated on CTA source images using the cortical vein opacification score (COVES) on the side of the stroke lesion (Jansen et al., 2018). Scores are assigned based on the imaging conditions of the Labbe vein, the sphenoparietal sinus, and the superficial middle cerebral vein (0 = no opacification; 1 = moderate; 2 = complete), with a total score ranging from 0 to 6. VO status was classified as “good” (COVES 3 ~ 6) or “impaired” (COVES 0 ~ 2).

The DWI infarct core volume was measured manually on average diffusion coefficient (ADC) maps, defined as the area with an ADC value below 620 × 10^−6^ mm^2^/s (Cimflova et al., 2021; Cohen et al., 2026). Two experienced researchers performed the measurements independently, and the average values were used for analysis.

### 2.4 Group definition

Patients were classified into four groups based on FVHs (high vs. low) and COVES (good vs. impaired): Type I (good coupling): high FVHs with good VO; Type II (arteriovenous mismatch): high FVHs with impaired VO; Type III (hypoperfusion type): low FVHs with good VO; Type IV (arteriovenous failure): low FVHs with impaired VO. G02 was defined as a binary variable derived from the four-group classification, with G02 = 1 indicating Type II (arteriovenous mismatch) and G02 = 0 indicating all other types. Representative imaging examples are shown in Figure 2.

**Figure 2:**
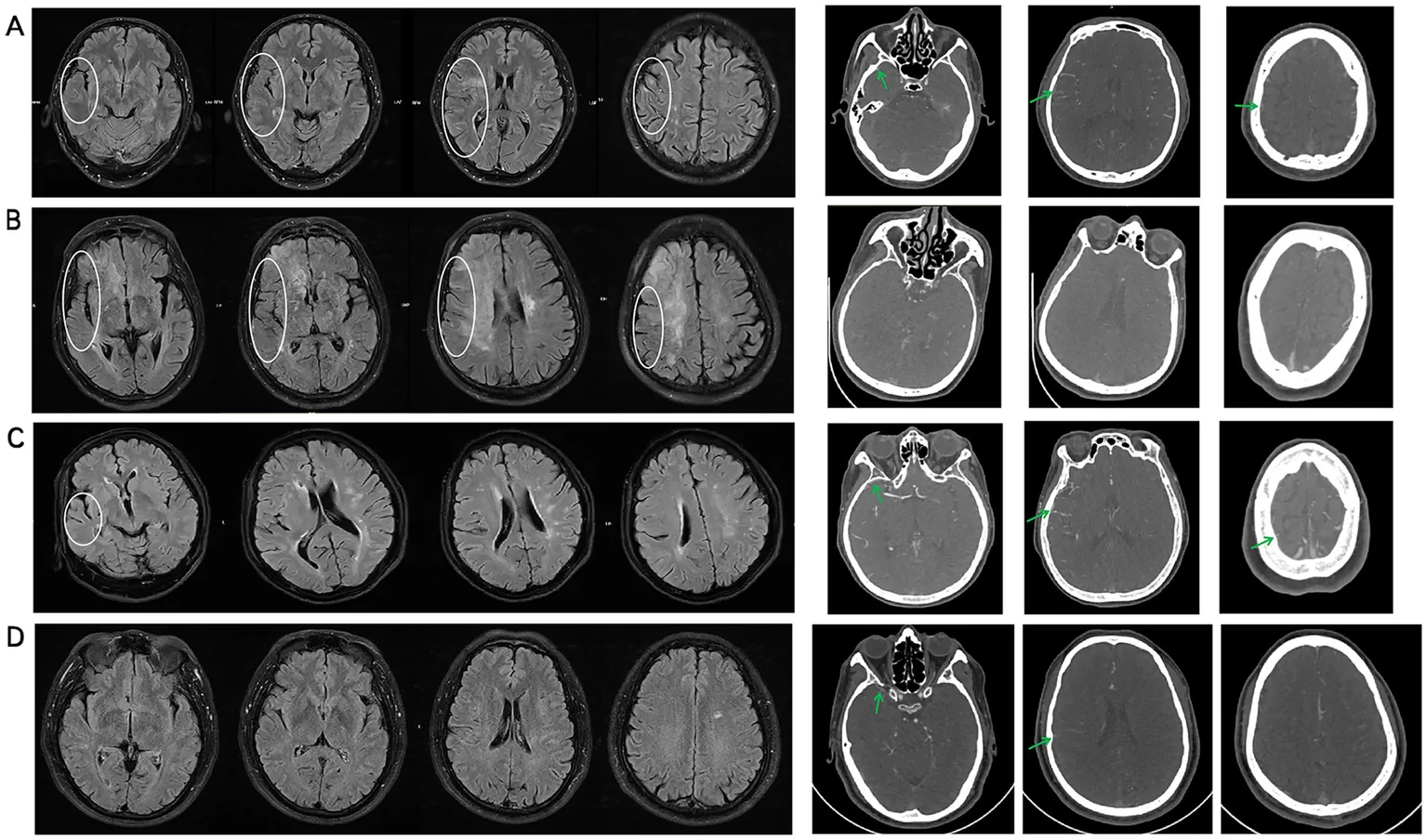
Representative images of the four arteriovenous coupling types. Each row shows a typical case from one of the four groups: **(A)** Type I (good coupling), **(B)** Type II (arteriovenous mismatch), **(C)** Type III (hypoperfusion type), and **(D)** Type IV (arteriovenous failure). Left column: FLAIR images used for FVHs assessment; white circles indicate FLAIR vascular hyperintensities. Right column: CTA images used for COVES scoring; green arrows indicate opacified cortical veins reflecting venous outflow status.

### 2.5 Statistical analysis

All statistical analyses were performed using SPSS version 26.0 (IBM Corp., Armonk, NY, USA). Continuous variables were expressed as mean ± standard deviation or median with interquartile range, and group comparisons were made using one-way analysis of variance (ANOVA) or the Kruskal–Wallis test, as appropriate. Categorical variables were presented as counts with percentages and compared using the chi-square test or Fisher’s exact test.

Multivariate logistic regression was used to identify independent predictors of a favorable 90-day outcome, with the arteriovenous coupling classification as the primary independent variable (with Type I as the reference). Covariates included age, admission NIHSS score, DWI volume, atrial fibrillation, mannitol use, and antiplatelet therapy. Results are reported as odds ratios (*OR*) with 95% *CI*. Candidate variables were selected *a priori* based on clinical relevance and prior literature. To avoid overfitting given the moderate sample size, we prioritized variables consistently reported as independent predictors of outcome, including age, admission NIHSS, DWI volume, and atrial fibrillation. Mannitol use and antiplatelet therapy were included as covariates to adjust for potential confounding. Door-to-MRI time was not included as it reflects workflow rather than disease severity. ASPECTS and occlusion site were not included due to high collinearity with DWI volume, which provides more granular quantitative information.

Several sensitivity analyses were performed: (1) using a binary variable (G02) to indicate the presence of arteriovenous mismatch, instead of the four-group classification; (2) adding an interaction term between FVHs and VO; (3) excluding patients who underwent decompressive craniectomy; and (4) redefining favorable outcome as mRS 0 ~ 1 and mRS 0 ~ 3. Subgroup analyses were performed according to DWI volume (< 30 mL vs. ≥ 30 mL). For predictive model development, two models were assessed using five-fold cross-validation. The entire dataset (*n* = 152) was randomly partitioned into five equal-sized subsets using stratified sampling to preserve the distribution of the outcome variable across folds. In each iteration, four folds were used as the training set to fit the model, and the remaining fold served as the validation set. This process was repeated five times, with each fold serving as the validation set once. The final performance metrics were calculated as the average of the five validation results. The base model included admission NIHSS score, DWI volume, and age; the full model added G02 to the base variables (with Type II coded as 1 and all others as 0). Model performance was evaluated using the area under the receiver operating characteristic curve (AUC) with bootstrap 95% CI, the net reclassification improvement index (NRI), and the integrated discrimination improvement index (IDI). Receiver operating characteristic (ROC) curves, calibration curves, and decision curves were plotted.

All tests were two-sided, and a *p*-value < 0.05 was considered statistically significant.

## 3 Results

### 3.1 Baseline characteristics

A total of 152 patients were included in this study. The four groups comprised 42 patients (27.6%) in Type I, 28 (18.4%) in Type II, 46 (30.3%) in Type III, and 36 (23.7%) in Type IV. Baseline characteristics are summarized in Table 1. Significant differences were observed across the four groups in age, the proportion of atrial fibrillation, door-to-MRI time, admission NIHSS score, neutrophils count, NLR, HDL and DWI infarct volume (all *p* < 0.05). Patients in Type II had the highest mean age (72.1 ± 5.9 years), the highest median NIHSS score (18), the largest median DWI volume (72.0 mL), and the highest proportion of atrial fibrillation (39.3%). Other laboratory parameters and treatment measures were balanced across groups (all *p* > 0.05).

**Table 1:** Baseline characteristics of the study population (by arteriovenous coupling groups).

Variable	Overall (*n* = 152)	Type I (*n* = 42)	Type II (*n* = 28)	Type III (*n* = 46)	Type IV (*n* = 36)	*p* value
Age (years), mean ± SD	68.3 ± 5.9	66.2 ± 5.1	72.1 ± 5.9	67.8 ± 5.1	69.0 ± 5.4	<0.001
Male, *n* (%)	64 (42.1)	18 (42.9)	13 (46.4)	16 (34.8)	17 (47.2)	0.654
Hypertension, *n* (%)	96 (63.2)	29 (69.0)	17 (60.7)	30 (65.2)	20 (55.6)	0.642
Diabetes mellitus, *n* (%)	59 (38.8)	19 (45.2)	10 (35.7)	17 (37.0)	13 (36.1)	0.796
Atrial fibrillation, *n* (%)	30 (19.7)	1 (2.4)	11 (39.3)	10 (21.7)	8 (22.2)	0.002
Coronary artery disease, *n* (%)	27(17.8)	10 (23.8)	6 (21.4)	7 (15.2)	4 (11.1)	0.457
Prior stroke, *n* (%)	18 (11.8)	6 (14.3)	1 (3.6)	5 (10.9)	6 (16.7)	0.404
Smoking history, *n* (%)	39 (25.7)	7 (16.7)	8 (28.6)	10 (21.7)	14 (38.9)	0.134
Systolic BP (mmHg)	146.6 ± 12.4	147.3 ± 11.5	145.5 ± 12.2	146.6 ± 12.8	146.9 ± 13.6	0.944
Diastolic BP (mmHg)	84.9 ± 9.3	85.9 ± 8.1	84.6 ± 10.0	84.8 ± 9.6	84.2 ± 10.0	0.884
Door-to-MRI time (h)	16.5 (13.5 ~ 20.0)	14.9 (13.6 ~ 16.9)	18.7 (16.6 ~ 20.1)	15.5 (14.3 ~ 17.5)	17.1(15.9 ~ 19.2)	<0.001
Admission NIHSS, median (IQR)	15.0 (12.0 ~ 17.2)	12.5 (10.0 ~ 15.0)	18.0 (15.0 ~ 20.0)	14.0 (12.0 ~ 16.0)	16.0 (12.0 ~ 18.0)	<0.001
White blood cells (×10^9^/L),	7.8 (7.0 ~ 8.5)	8.1 (7.1 ~ 8.8)	7.8 (6.7 ~ 8.7)	7.2 (6.6 ~ 8.3)	7.9 (6.7 ~ 9.1)	0.351
Ne (×10^9^/L)	6.2 (5.5 ~ 7.0)	6.1 (4.1 ~ 8.2)	7.7 (6.4 ~ 10.7)	6.4 (4.3 ~ 7.7)	6.9 (5.0 ~ 9.0)	0.018
Lym (×10^9^/L)	1.4 (1.2 ~ 1.6)	1.5 (1.1 ~ 2.1)	1.6 (1.2 ~ 2.1)	1.4 (1.0 ~ 1.9)	1.5 (1.2 ~ 1.8)	0.834
NLR	4.5 (3.5 ~ 6.0)	4.0 (3.3 ~ 4.6)	5.6 (5.0 ~ 6.0)	4.1 (3.5 ~ 5.0)	5.0 (4.1 ~ 5.6)	<0.001
Hemoglobin (g/L)	135.7 ± 11.7	138.9 ± 11.1	136.1 ± 12.2	133.3 ± 12.1	134.8 ± 11.2	0.161
Platelets (×10^9^/L)	205.6 ± 35.3	208.0 ± 37.3	199.2 ± 36.0	202.4 ± 37.3	212.1 ± 29.5	0.446
Hs-CRP (mg/L), median (IQR)	5.0 (3.5 ~ 7.0)	6.0 (4.5 ~ 7.7)	5.3 (2.8 ~ 6.8)	6.1 (4.3 ~ 8.2)	5.8 (3.9 ~ 7.0)	0.269
D-dimer (mg/L), median (IQR)	0.4 (0.3 ~ 0.6)	0.4 (0.3 ~ 0.6)	0.3 (0.2 ~ 0.4)	0.4 (0.2 ~ 0.5)	0.5 (0.3 ~ 0.6)	0.169
Blood glucose (mmol/L)	6.5 (5.8 ~ 7.2)	6.8 (5.8 ~ 7.4)	6.0 (5.6 ~ 7.0)	6.2 (5.4 ~ 7.2)	6.8 (6.0 ~ 7.6)	0.345
HbA1c (%), median (IQR)	6.0 (5.6 ~ 6.4)	6.0 (5.5 ~ 6.3)	6.0 (5.6 ~ 6.3)	6.0 (5.6 ~ 6.5)	6.1 (5.7 ~ 6.5)	0.881
TC (mmol/L)	4.3 ± 0.7	4.4 ± 0.6	4.3 ± 0.6	4.3 ± 0.6	4.2 ± 0.8	0.464
TG (mmol/L)	1.4 (1.1 ~ 1.7)	1.5 (1.0 ~ 1.8)	1.7 (0.9 ~ 1.9)	1.4 (1.1 ~ 1.8)	1.6 (1.1 ~ 1.8)	0.884
HDL (mmol/L)	1.1 ± 0.2	1.0 ± 0.2	1.2 ± 0.2	1.1 ± 0.2	1.2 ± 0.2	0.042
LDL (mmol/L)	2.6 ± 0.6	2.5 ± 0.6	2.5 ± 0.6	2.5 ± 0.5	2.5 ± 0.6	0.986
Cr (μmol/L)	74.6 ± 12.2	73.3 ± 11.9	71.9 ± 12.6	76.8 ± 9.5	75.3 ± 14.8	0.330
Ccr (mL/min)	82.3 (71.6 ~ 93.8)	78.1 (66.6 ~ 92.0)	83.8 (64.0 ~ 90.6)	84.2 (71.3 ~ 99.3)	82.7 (77.7 ~ 93.2)	0.691
Hcy (μmol/L), median (IQR)	12.0 (10.0 ~ 14.0)	11.8 (9.9 ~ 14.0)	11.1 (9.9 ~ 13.6)	11.6 (9.8 ~ 13.5)	12.6 (11.0 ~ 14.4)	0.318
M1 occlusion, n (%)	118 (77.6)	32 (76.2)	23 (82.1)	34 (73.9)	29 (80.6)	0.820
Left hemisphere, n (%)	61 (40.1)	13 (31.0)	13 (46.4)	22 (47.8)	13 (36.1)	0.346
DWI volume (mL), median (IQR)	50.5 (36.0 ~ 66.0)	33.0 (23.2 ~ 42.0)	72.0 (61.0 ~ 79.2)	42.0 (36.0 ~ 55.8)	60.5 (51.5 ~ 70.2)	<0.001
ASPECTS, median (IQR)	8 (7 ~ 9)	8 (6 ~ 10)	8 (7 ~ 8)	8 (6 ~ 9)	8 (7 ~ 9)	0.435
Mannitol use, n (%)	72 (47.4)	20 (47.6)	12 (42.9)	22 (47.8)	18 (50.0)	0.954
Antiplatelet therapy, n (%)	131 (86.2)	38 (90.5)	24 (85.7)	39 (84.8)	30 (83.3)	0.807
Statin therapy, n (%)	131 (86.2)	35 (83.3)	23 (82.1)	41 (89.1)	32 (88.9)	0.747
Decompressive craniectomy, n (%)	15 (9.9)	1 (2.4)	5 (17.9)	5 (10.9)	4 (11.1)	0.189

MRI, magnetic resonance imaging; NIHSS, National Institutes of Health Stroke Scale; Ne, neutrophil count; Lym, lymphocyte count; NLR, neutrophil-to-lymphocyte ratio; hs-CRP, high-sensitivity C-reactive protein; HbA1c, glycated hemoglobin; TC, total cholesterol; TG, triglycerides; HDL, high-density lipoprotein; LDL, low-density lipoprotein; Cr, creatinine; Ccr, creatinine clearance rate; Hcy, homocysteine; DWI, diffusion-weighted imaging; ASPECTS, Alberta Stroke Program Early CT Score.

### 3.2 Multivariate logistic regression analysis

After adjusting for age, admission NIHSS score, DWI volume, atrial fibrillation, mannitol use, and antiplatelet therapy, Type II (arteriovenous mismatch) remained an independent negative predictor of a favorable 90-day outcome (*OR* = 0.041, 95% *CI* 0.003 ~ 0.506, *p* = 0.013). To assess the stability of the effect estimate for Type II, we performed Bootstrap resampling with 1000 iterations. The bias-corrected *OR* was 0.212 (95% *CI* 0.106–0.497), confirming the direction and consistency of the original finding. Compared with Type I, Type III yielded an *OR* of 0.288 (95% *CI* 0.090 ~ 0.919, *p* = 0.035), while Type IV showed no statistically significant difference (*p* = 0.710). Age (*OR* = 0.906, *p* = 0.034) and atrial fibrillation (*OR* = 0.030, *p* = 0.001) were also significantly associated with a lower likelihood of favorable outcome. Results are presented in Table 2.

**Table 2:** Multivariate logistic regression analysis (independent predictors of 90-day good outcome).

Variable	*β* coefficient	*OR* (95% *CI*)	*p* value
Type II vs. Type I (arteriovenous mismatch)	−3.186	0.041 (0.003 ~ 0.506)	0.013
Type III vs. Type I	−1.244	0.288 (0.090 ~ 0.919)	0.035
Type IV vs. Type I	0.291	1.337 (0.289 ~ 6.178)	0.710
Age (per 1 ~ year increase)	−0.099	0.906 (0.827 ~ 0.992)	0.034
Atrial fibrillation	−3.521	0.030 (0.003 ~ 0.254)	0.001
Admission NIHSS (per 1 ~ point increase)	−0.102	0.903 (0.780 ~ 1.045)	0.171
DWI volume (per 1 mL increase)	−0.023	0.977 (0.949 ~ 1.006)	0.121
Mannitol use	0.655	1.926 (0.779 ~ 4.762)	0.156
Antiplatelet therapy	0.915	2.498 (0.736 ~ 8.480)	0.142

NIHSS, National Institutes of Health Stroke Scale; DWI, diffusion-weighted imaging.

### 3.3 Sensitivity analyses

To test the robustness of the findings, several sensitivity analyses were performed, with results summarized in Table 3, separately shown in Supplementary Tables S2–S5.

**Table 3:** Summary of sensitivity analyses.

Sensitivity analysis	OR (95% CI)	*p* value
Binary high-risk group (G02)	0.055 (0.006 ~ 0.503)	0.010
Interaction model (FVHs and VO)	0.208 (0.051 ~ 0.853)	0.025
Excluding decompressive craniectomy	0.032 (0.002 ~ 0.446)	0.011
Loose good outcome definition (mRS 0 ~ 3)	0.259 (0.045 ~ 1.492)	0.131

FVHs, fluid-attenuated inversion recovery vascular hyperintensities; VO, venous outflow; mRS, modified Rankin scale.

#### 3.3.1 Binary high-risk grouping

When G02 was entered as a binary variable (arteriovenous mismatch vs. non-arteriovenous mismatch), the results remained consistent with the main analysis (*OR* = 0.055, 95% *CI* 0.006 ~ 0.503, *p* = 0.010) (Supplementary Table S2).

#### 3.3.2 Interaction analysis

The interaction term between FVHs and VO was significant (*OR* = 0.208, 95% *CI* 0.051 ~ 0.853, *p* = 0.025), suggesting a synergistic effect between FVHs and venous status (Supplementary Table S3). Stratified analysis indicated that in patients with impaired VO, FVHs showed a stronger negative association with favorable outcome (approximately 79% reduction in the odds of good outcome per one-grade increase in FVHs), whereas this association was weaker in those with preserved venous outflow (*OR* close to 1). This suggests that VO impairment amplifies the adverse prognostic value of FVHs.

#### 3.3.3 Excluding decompressive craniectomy patients

After excluding the 14 patients who underwent decompressive craniectomy (*n* = 137), Type II remained significantly associated with poor outcome (*OR* = 0.032, 95% *CI* 0.002 ~ 0.446, *p* = 0.011), supporting the robustness of the main findings (Supplementary Table S4).

#### 3.3.4 Redefining favorable outcome

When favorable outcome was defined more strictly as mRS 0 ~ 1, the model failed to converge because no patient in Type II achieved a good outcome under this definition. When defined more broadly as mRS 0 ~ 3, the *OR* for Type II was 0.259 (95% *CI* 0.045 ~ 1.492, *p* = 0.131), showing a consistent direction albeit with attenuated effect, as expected (Supplementary Table S5). These sensitivity analyses collectively support the robustness of the primary conclusions.

### 3.4 Subgroup analysis

Patients were divided according to the median DWI volume (30 mL) into a small infarct core group (Small, *n* = 24) and a large infarct core group (Large, *n* = 128). The small group was too small for multivariate modeling; therefore, only descriptive statistics were presented for this subgroup. In the large group, multivariate logistic regression showed that Type II remained a strong predictor of poor outcome (*OR* = 0.026, 95% *CI* 0.002 ~ 0.301, *p* = 0.003), consistent with the overall cohort findings (Supplementary Table S6), indicating that the negative prognostic effect of arteriovenous mismatch was particularly pronounced in patients with larger infarct cores.

### 3.5 Predictive model performance

The AUCs of the predictive models from five-fold cross-validation are shown in Table 4. The base model (NIHSS + DWI volume + age) achieved an AUC of 0.759 (95% *CI* 0.704 ~ 0.849). Adding G02 to the base model improved the AUC to 0.780 (95% *CI* 0.730 ~ 0.870), with a difference of 0.021 (95% *CI* 0.002 ~ 0.046, *p* = 0.0325 by DeLong’s test). The NRI was 0.0305 and the IDI was 0.0348, indicating a modest but positive reclassification improvement with the addition of G02. ROC curves, calibration curves, and decision curves are shown in Figure 3 and Supplementary Figures S1, S2. The decision curve analysis demonstrated that the full model provided higher net clinical benefit across the entire range of threshold probabilities.

**Table 4:** Discriminative performance of predictive models and individual predictors for 90-day good outcome (5-fold cross-validation).

Model / predictor	AUC	95% *CI*
Age	0.687	0.604 ~ 0.770
NIHSS	0.632	0.553 ~ 0.724
DWI volume	0.714	0.631 ~ 0.789
G02 (arteriovenous mismatch)	0.651	0.614 ~ 0.725
Base model (NIHSS+DWI + Age)	0.759	0.704 ~ 0.849
Full model (Base+G02)	0.780	0.730 ~ 0.870

NIHSS, National Institutes of Health Stroke Scale; DWI, diffusion-weighted imaging.

**Figure 3:**
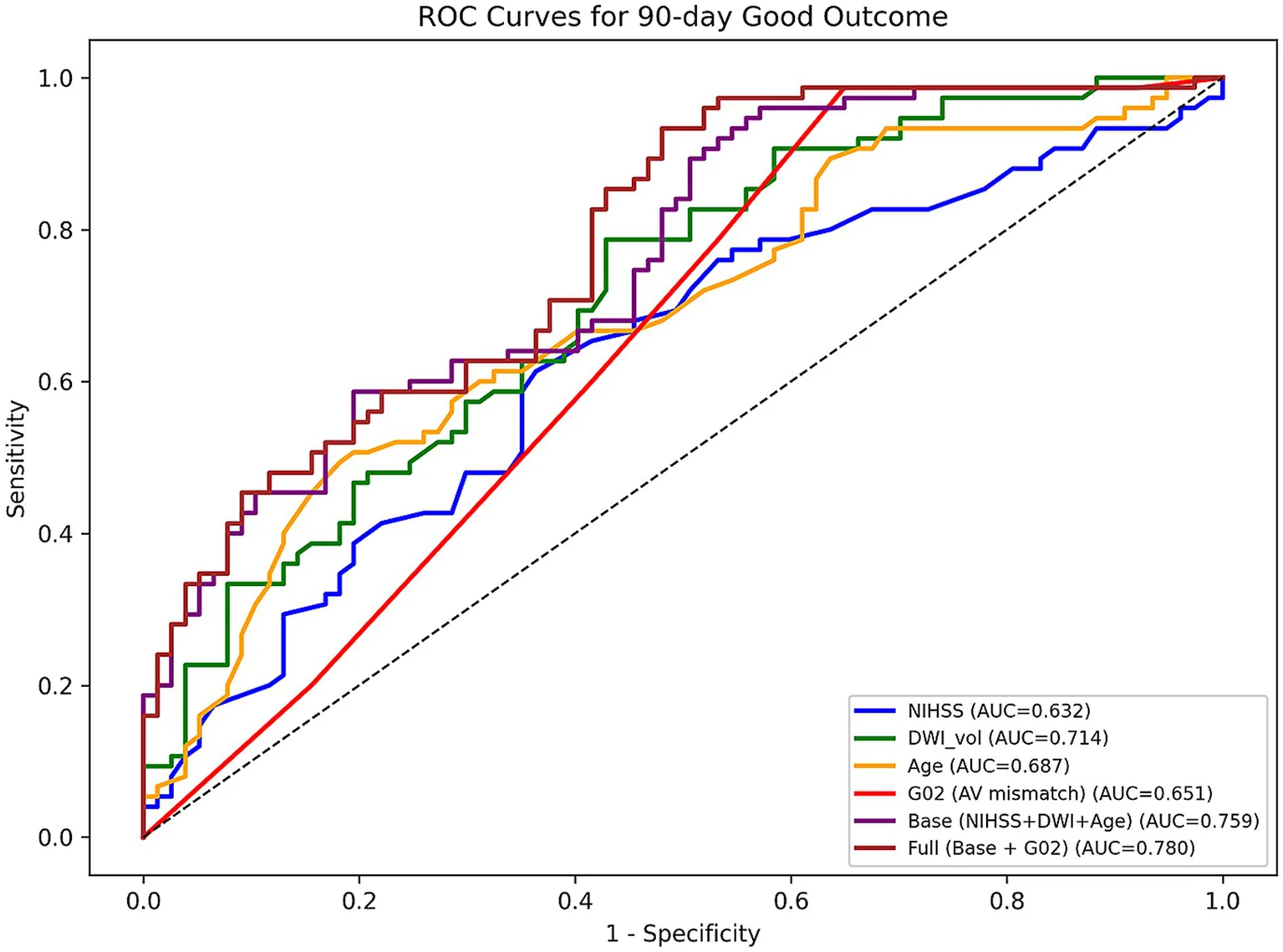
ROC analysis for predicting 90-day good outcome. ROC, receiver operating characteristic; NIHSS, National Institutes of Health Stroke Scale; AUC, area under the curve; DWI, diffusion-weighted imaging.

## 4 Discussion

Clinical outcomes vary considerably among patients with acute MCA occlusion who do not receive reperfusion therapy. This variability is closely linked to collateral compensatory capacity, cerebral autoregulatory function, and microcirculatory status (Mondal et al., 2025). Collateral circulation plays a critical role in preserving the ischemic penumbra (Almeida et al., 2026), and well-developed collaterals can extend the window of ischemic tolerance and limit infarct expansion (Arteaga et al., 2022). However, good collateral circulation alone is not sufficient to ensure a favorable outcome. Animal studies of cerebral ischemia have shown that microthrombi can be detected in small veins within 30 min of acute cerebral hypoperfusion (Zhang et al., 2012). Moreover, poor venous drainage in the ischemic territory has been associated with severe hemispheric symptoms and larger infarct volumes (Zhang et al., 2017; Zhang et al., 2023). Collectively, these observations suggest that cerebral veins are critical for maintaining arterial perfusion and neurological function after ischemic stroke. Therefore, relying solely on arterial collateral assessment without considering VO status is unlikely to provide a complete picture of prognosis in these patients who do not undergo reperfusion therapy.

In this study, we used a combined MRI and CTA approach to evaluate arteriovenous status. FVHs were assessed on MRI-FLAIR sequences, which are sensitive to slow flow and can reflect the presence of collateral flow without the need for exogenous contrast agents (Lee et al., 2022). VO was evaluated on CTA, which provides clear visualization of contrast-filled venous structures and allows assessment of the anatomic patency of the VO tract (Zhang et al., 2023). Together, these two imaging modalities offer complementary information: FLAIR captures the functional aspect of collateral compensation, while CTA depicts the structural aspect of venous drainage. A recent study combining CTA-based COVES with FLAIR hyperintense acute reperfusion marker demonstrated an association between VO status and parenchymal injury (Zamarud et al., 2025). Building on that work, the present study extends the concept by testing whether VO status may modulate the prognostic value of collateral circulation in patients who did not receive reperfusion therapy.

In the study, we combined FVHs with VO assessment to systematically evaluate the predictive value of arteriovenous coupling status for 90-day outcomes. Our main findings are as follows. First, arteriovenous mismatch (Type II: high FVH with impaired VO) emerged as an independent predictor of favorable 90-day outcome, with an approximately 96% reduction in the odds of good prognosis compared with good coupling (Type I: high FVHs with preserved VO). Second, adding the arteriovenous mismatch indicator to conventional clinical variables (NIHSS, DWI volume, and age) moderately improved prognostic performance, with AUC increasing from 0.759 to 0.780. Third, this incremental effect was more pronounced in patients with large infarct cores (DWI volume ≥ 30 mL). These observations are in line with recent reports showing that unfavorable VO predicts poor prognosis (Kelani et al., 2025; Gu et al., 2025; Mei et al., 2025). The most notable finding of our study is that the prognostic value of FVHs is conditional upon VO status. In patients with preserved VO (Type I), abundant FVHs was associated with favorable outcomes, consistent with the conventional view of FVHs as a marker of collateral compensation. In patients with impaired VO (Type II), however, abundant FVHs were paradoxically associated with poor outcomes. This indicates that abundant FVHs do not invariably indicate a good prognosis; its clinical significance depends on whether VO is patent (Tong et al., 2018). Given the modest AUC improvement (0.021) and the relatively small NRI (0.0305) and IDI (0.0348) values, the incremental value of G02 for individualized outcome prediction is limited. Instead, the arteriovenous mismatch marker appears more suitable for risk stratification, helping to identify patients with elevated baseline risk who may warrant more intensive monitoring or treatment. The non-significant difference between Type IV and Type I (*OR* = 1.337, *p* = 0.710) is likely due to limited subgroup size (*n* = 36) and should not be interpreted as biological equivalence. Compared with Type II, Type IV patients had lower NIHSS scores and smaller DWI volumes, suggesting less severe disease. The absence of abundant FVHs implies limited collateral inflow without the added venous congestion seen in Type II. Thus, the arteriovenous mismatch phenotype appears more detrimental than the absence of both.

This observation offers a fresh perspective on the inconsistency of previous FVHs studies. Some earlier researches have suggested that FVHs indicates good collaterals and favorable outcomes, while others have associated FVHs with poor prognosis (Jiang et al., 2019; Zeng et al., 2021; Derraz et al., 2022). Xiang et al. reported that combining FVHs with venous markers (asymmetric prominent venous signs) can enhance the prognostic prediction ability for patients with acute ischemic stroke, with an AUC of 0.921, their work partially supports our observation that the prognostic value of FVHs may be modulated by VO status (Xiang et al., 2023). When VO is preserved, FVHs reflects effective collateral input and correlates with good prognosis. When VO is impaired, FVHs reflects collateral flow that cannot be effectively drained, leading to ineffective perfusion and poor outcomes. Thus, combined assessment of FVHs and VO status enables more accurate prediction of prognosis and provides a mechanistic explanation for the variable prognostic significance of FVHs reported in the literature.

Our study has several clinical and scientific implications. First, it provides a straightforward imaging-based risk stratification tool for patients with acute MCA occlusion who present beyond the time window, have contraindications to reperfusion therapy, or are managed conservatively. In such patients, combined evaluation of FVHs and VO allows rapid identification of the high-risk phenotype (Type II), who may benefit from more intensive monitoring, early osmotic therapy, or even evaluation for prophylactic decompressive craniectomy. Second, it highlights that VO status may be an important factor influencing the clinical benefit derived from collateral circulation, underscoring the need to assess venous status alongside collateral evaluation. Finally, it points to new directions for future research, including therapeutic strategies targeting venous outflow impairment, such as osmotic therapy, venous sinus angioplasty, or avoidance of excessive blood pressure reduction, which may improve outcomes in this patient subset.

Several limitations should be acknowledged. The retrospective, single-center design may introduce selection bias, and our findings require prospective multicenter validation. The modest sample size limited the statistical power, particularly for Type II (*n* = 28) and Type IV (*n* = 36). The wide *CI* around the original *OR* estimate for Type II (*n* = 28) likely reflects the small size of this subgroup, which was supported by Bootstrap validation that confirmed the direction of the association while attenuating the effect estimate. The non-significant difference between Type IV and Type I may also be related to the limited sample size of the Type IV subgroup. In addition, the small infarct core subgroup (<30 mL, *n* = 24) was not suitable for multivariable analysis; therefore, those findings should be interpreted as exploratory and hypothesis-generating. Cortical venous outflow was assessed on single-phase CTA without dynamic flow information, and the combined MRI and CTA approach may not be feasible in centers without access to both modalities, potentially limiting broad applicability. Additionally, door-to-MRI time was not standardized in the present center and varied across patients, largely due to operational factors such as MRI scheduling, patient transport, and cooperation during scanning. This variability may have influenced the imaging findings, particularly DWI volume, which could in turn have affected the results. Although the observed differences in DWI volume across groups were primarily attributed to disease severity, the possibility that variations in imaging timing contributed to these findings cannot be completely excluded. Patients receiving reperfusion therapy were excluded, limiting generalizability to those undergoing thrombectomy or thrombolysis. Long-term outcome data were not available. Additionally, we did not apply an individualized interpretative framework for FVHs, such as distinguishing proximal from distal FVHs or its differing implications in stenosis versus occlusion (Lv et al., 2023), which may have introduced heterogeneity in our grouping and should be considered when interpreting our findings.

## 5 Conclusion

Arteriovenous mismatch (high FVHs with impaired VO) independently predicts poor 90-day outcome in non-reperfused acute MCA occlusion. Its incorporation into conventional risk models may improve risk stratification, suggesting that VO status may play an important role in modulating the clinical benefit of collateral flow. This finding supports combined assessment of arterial and venous circulation in stroke risk stratification.

## Data Availability

The raw data supporting the conclusions of this article will be made available by the authors, without undue reservation.
