# Supplementary material for: Combined assessment of FLAIR vascular hyperintensities and cortical venous outflow for outcome prediction in acute middle cerebral artery occlusion
Source: Front Hum Neurosci. 2026 Sep 29;20:1953695. doi: 10.3389/fnhum.2026.1953695 (PMC13657956; doi:10.3389/fnhum.2026.1953695)
Supplement: Supplementary file 1 [file Data_Sheet_1.DOCX]

Table S1. Detailed MRI parameters

| Sequence | TR/TE/TI (ms) | FOV (mm) | Matrix | Slice thickness (mm) | Slice gap (mm) |
| --- | --- | --- | --- | --- | --- |
| T1‑weighted | 550 / 12 | 320 × 320 | 256 × 256 | 5 | 1 |
| T2‑weighted | 8070 / 69 | 320 × 276 | 256 × 256 | 5 | 1 |
| T2‑FLAIR | 9000 / 105 / 2500 | 240 × 240 | 256 × 192 | 5 | 1 |
| DWI | 2500 / 76 | 240 × 240 | 192 × 192 | 5 | 1 |

TR, repetition time; TE, echo time; TI, inversion time; FOV, field of view; FLAIR, fluid-attenuated inversion recovery; DWI, diffusion‑weighted imaging.

Table S2. Binary high‑risk group (G02) logistic regression for 90‑day good outcome

| Variable | *β* coefficient | *OR* (95% *CI*) | *P* value |
| --- | --- | --- | --- |
| G02 (high-risk vs. non-high-risk) | -2.892 | 0.055 (0.006~0.503) | 0.010 |
| Age | -0.089 | 0.915 (0.842~0.993) | 0.037 |
| Atrial fibrillation | -3.556 | 0.029 (0.003~0.230) | 0.001 |
| Admission NIHSS score | -0.075 | 0.928 (0.820~1.050) | 0.238 |
| Mannitol use | 0.652 | 1.919 (0.805~4.575) | 0.141 |
| Antiplatelet therapy | 0.823 | 2.278 (0.714~7.271) | 0.165 |
| DWI volume | -0.019 | 0.981 (0.957~1.005) | 0.123 |

NIHSS, National Institutes of Health Stroke Scale; DWI, diffusion‑weighted imaging.

Table S3. Interaction analysis (FHVs × VO)

| Variable | *β* coefficient | *OR* (95% *CI*) | *P* value |
| --- | --- | --- | --- |
| FHVs | 0.356 | 1.428 (0.867~2.348) | 0.161 |
| VO | 2.111 | 8.255 (1.878~36.27) | 0.005 |
| FHVs and VO | -1.570 | 0.208 (0.076~0.569) | 0.002 |
| Age | -0.106 | 0.899 (0.823~0.982) | 0.017 |
| Atrial fibrillation | -3.530 | 0.029 (0.003~0.250) | 0.001 |
| Admission NIHSS score | -0.119 | 0.888 (0.771~1.022) | 0.098 |
| DWI volume | -0.026 | 0.975 (0.947~1.003) | 0.084 |
| Mannitol use | 0.601 | 1.823 (0.753~4.416) | 0.183 |
| Antiplatelet therapy | 0.793 | 2.209 (0.676~7.214) | 0.189 |

FHVs, fluid-attenuated inversion recovery vascular hyperintensities; VO, venous outflow;

NIHSS, National Institutes of Health Stroke Scale; DWI, diffusion‑weighted imaging.

Table S4. Excluding decompressive craniectomy patients (*n*=137)

| Variable | *β* coefficient | *OR* (95% *CI*) | *P* value |
| --- | --- | --- | --- |
| Type II vs. Type I | -3.460 | 0.031 (0.002~0.446) | 0.011 |
| Type III vs. Type I | -1.391 | 0.249 (0.069~0.898) | 0.034 |
| Type IV vs. Type I | 0.068 | 1.070 (0.200~5.725) | 0.937 |
| Age | -0.112 | 0.894 (0.811~0.985) | 0.023 |
| Atrial fibrillation | -3.796 | 0.022 (0.002~0.203) | 0.001 |
| Admission NIHSS score | -0.089 | 0.915 (0.783~1.069) | 0.259 |
| DWI volume | -0.012 | 0.988 (0.957~1.020) | 0.461 |
| Mannitol use | 0.588 | 1.800 (0.679~4.770) | 0.237 |
| Antiplatelet therapy | 1.202 | 3.327 (0.948~11.68) | 0.060 |

NIHSS, National Institutes of Health Stroke Scale; DWI, diffusion‑weighted imaging.

Table S5. Loose good outcome (mRS 0~3)

| Variable | *β* coefficient | *OR* (95% *CI*) | *P* value |
| --- | --- | --- | --- |
| Type II vs. Type I | -1.352 | 0.259 (0.045~1.492) | 0.131 |
| Type III vs. Type I | -0.887 | 0.412 (0.123~1.377) | 0.150 |
| Type IV vs. Type I | -0.114 | 0.892 (0.203~3.930) | 0.880 |
| Age | -0.055 | 0.946 (0.878~1.020) | 0.151 |
| Atrial fibrillation | -1.222 | 0.295 (0.110~0.793) | 0.016 |
| Admission NIHSS score | -0.055 | 0.947 (0.837~1.071) | 0.385 |
| DWI volume | -0.027 | 0.974 (0.948~1.001) | 0.059 |
| Mannitol use | 0.331 | 1.392 (0.628~3.086) | 0.416 |
| Antiplatelet therapy | 1.159 | 3.185 (1.058~9.586) | 0.039 |

NIHSS, National Institutes of Health Stroke Scale; DWI, diffusion‑weighted imaging.

Table S6. Large infarct core group (DWI volume ≥30 mL, *n*=128)

| Variable | *β* coefficient | *OR* (95% *CI*) | *P* value |
| --- | --- | --- | --- |
| Type II vs. Type I | -3.639 | 0.026 (0.002~0.301) | 0.003 |
| Type III vs. Type I | -1.237 | 0.290 (0.075~1.122) | 0.073 |
| Type IV vs. Type I | 0.017 | 1.017 (0.219~4.724) | 0.982 |
| Age | -0.078 | 0.925 (0.839~1.020) | 0.118 |
| Atrial fibrillation | -3.801 | 0.022 (0.003~0.198) | 0.001 |
| Admission NIHSS score | -0.122 | 0.886 (0.752~1.041) | 0.141 |
| Mannitol use | 0.476 | 1.610 (0.604~4.292) | 0.343 |
| Antiplatelet therapy | 1.355 | 3.877 (1.001~15.02) | 0.050 |

NIHSS, National Institutes of Health Stroke Scale.

Figure S1. Calibration curves for the base and full models.

The full model showed better agreement between predicted and observed probabilities.

Figure S2. Decision curves for the base and full models.

The full model provided greater net clinical benefit across the threshold range.
